# Operative public values as a tool for healthcare decisions: the social value and clinical criteria of triage

**DOI:** 10.1186/s13010-022-00124-2

**Published:** 2022-09-28

**Authors:** Luis Cordeiro-Rodrigues

**Affiliations:** Department of Philosophy, Yuelu Academy, Hunan University, Changsha, 410082 China

**Keywords:** Triage, COVID-19, Clinal criteria, Social value, Racial and class bias, Pandemic, Public health, Operative public values

## Abstract

With the current pandemic, many scholars have contended that clinical criteria offer the best way to implement triage. Further, they dismiss the criteria of social value as a good one for triage. In this paper, I respond to refute this perspective. In particular, I present two sets of arguments. Firstly, I argue that the objections to the social value criteria they present apply to the clinical criteria they favor. Secondly, they exaggerate the negative aspects of the social value criteria, while I suggest it is reasonable to use this. I end the article by recommending how operative public values can be a good way to make triaging decisions.

## Background

The COVID-19 pandemic brought an unprecedented preoccupation with the ethics of triaging health resources [1]. This is unsurprising because while before COVID-19 the degree of triage was not so significant, the pandemic caused a massive scarcity of resources. Recent literature argues that the appropriate criteria for triage patients are clinal ones and uphold that social value is an inadequate criterion for triage [2]. In the context of triage, it is meant by the ‘social value criterion’ the use of the principle that individuals’ morality, moral status, and contributions to society ought to be the canon to evaluate whom to save first when there are not sufficient medical resources for everyone. In this comment, I contend that they neglect how their objections to the social value criteria apply to the clinical criteria they uphold and that the problems they point out for the social value criteria are overstated. In the next section, I summarize the argument of those who contend that social value is an inadequate criterion for triage. In the section after that, I offer my objections against their arguments. In the third section, I provide some positive reasons to endorse the social value criterion for triage.

I have previously defended that social value is a good criterion for triage [3, 4]. But the current paper differs from previous ones in at least five ways. Firstly, in contrast with previous work that was focused specifically on a specific social criterion – the social harmony one – in this article, I am defending social values in triage in general [3, 4]. Secondly, contrasting with the arguments made before, I do not think that the social value criterion needs to be universal in the sense of being the same in different cultures. Instead, I contend in this article that the operative public values (defined below) of a specific society ought to be the guidance for triage decisions for that society. Thirdly, in contrast with previous work, in this article, I defend the social value criterion by intertwining it with the duties of healthcare professionals to be aware of ethical issues in their profession and the relationship between public trust and health policy. Fourthly, while previous research has conceptualized clinical criteria as different from social criteria, in this article, I demonstrate that the former is, independently of their importance, insufficient for making decisions about health and are ultimately reducible to the latter. Fifthly, I explain that despite the incomplete information one may have about the social value it is still justified to use this criterion at least in situations of emergency ethics, such as a pandemic.

### The case for using clinical criteria instead of social value criteria in triage decisions

The main argument is that clinical criteria are the most objective set of criteria. As such, these are the best way to fulfil the ethical principles of equity and the greatest good, that ought to guide triage decisions. The principle of equity states that triage decisions should apply to all patients who may require intensive care, independent of being COVID-19 patients or not. The principle of the greatest good prescribes that those patients with the most favorable prognosis (most likely to survive and benefit from treatment), as well as those who will predictably have a shorter length of expected intensive care unit (ICU) admission, ought to be given priority unless they voluntarily refuse admission to an ICU [2].

With this groundwork in mind, Herreros, Gella, and Asua, for example, criticize the Spanish Society for Critical and Intensive Medicine and Coronary Units (SEMICYUC) for including less objective and ethically dubious criteria in triage decisions, and social value. The social value criterion proposed was that prioritization should be attributed according to the social contribution made by the individual to society. They object to this criterion with four arguments. Firstly, it is difficult to agree on the social value of an individual to society because this depends on a myriad of factors that are difficult to measure. This is an argument about wide disagreement in society regarding what aspects ought to be valued; this wide disagreement, they contend, is sufficient for not endorsing this criterion. Secondly, even if it was measurable, healthcare professionals are not trained or fit to make such an assessment. The lack of knowledge of such moral reasoning by healthcare professionals then suffices as a reason not to use social value as a criterion for triage. Thirdly, such a criterion could undermine social trust in the profession. Fourthly, this criterion could lead to arbitrary decision-making that discriminates against many collectives. In other words, the social value criterion allows discriminatory practices because of its vagueness, i.e., healthcare professionals can use it as they wish [2].

### Social value and the problem with clinical criteria for triage

In this section, I wish to present two kinds of objections against the previously raised arguments. I want to show that the objections raised against the social value criterion also apply to the clinical criteria. Hence, there is no reason to value clinical criteria over social value. Further, I wish to contend that this is not clear-cut that those objections are problematic as they uphold the social value criterion. Firstly, given the unexpected and recent impact of the pandemic, most scientific research on COVID-19 is ongoing, making it significantly challenging to have reliable criteria to decide who is most likely to benefit from treatment and survive. For example, it is unclear who has more probability of surviving if they receive treatment: an older, healthy individual aged over eighty or a young, obese, and diabetic one under the age of thirty? Surely, with time and research development, the clinical criteria may be more reliable, but currently, the evidence is dispersed and routinely finds new and contradictory variables on what factors make it most likely a person will survive.

Moreover, there will always be a high degree of uncertainty in clinical criteria, as so many factors are to consider. There is no clinical criterion that can inform healthcare professionals on what ought to be given priority; instead, the prioritization of, say, an older person over a diabetic young person is ultimately a moral evaluation of who ought to live. My point is that ultimately clinical criteria need to make a social/moral evaluation to decide. Therefore, not only it is the case that clinical criteria lack objectivity and information to make triaging decisions, making measures difficult, but also such criteria are insufficient in themselves to make a decision and they ultimately need to resort to moral/social criteria. For instance, the decision to use the clinical criterion of who is most likely to live is grounded on the normative (rather than clinical) idea that living more is better than living less.

The social value may be challenging to measure, but there are straightforward examples where it is not [4]. Pedophiles, rapists, arsonists, and convicts of other serious crimes have a low social value and this can be used as one of the criteria for triage. Of course, we may not have complete information on individuals’ social value, but this is also true about clinical-based decisions. Incomplete information is a problem and the more information the better so that healthcare decisions are morally justified. Indeed, much of the recent health informatics work focuses on providing the maximum accurate information possible to avoid medical errors [5, 6].

Nonetheless, I have elsewhere suggested a possible method for storing this kind of information and helping healthcare professionals to make decisions [3, 4]. The reality of health emergencies is that these require decisions under incomplete information. Analogous to a situation in a war where it is unrealistic to ask soldiers to consult all the information before deciding because there is a need to make fast judgments [7, 8], in a pandemic, many decisions ought to be made in a fast manner [9]. To make decisions with incomplete information is the only way to address moral questions in emergency ethics; hence, because we live in a non-ideal world, it is morally justified to make such decisions under pressure.

The fact that healthcare professionals are not fit or trained for such decision-making is a surprising claim that implies that they are merely technicians without ethical training. This is unlikely to be true, especially given that medicine is an enterprise with a significant moral aspect [10]. Moreover, any profession ought to receive ethical training relevant to their profession [11]. Medical schools seem to be required to train their professionals this way because these people will be dealing with life and death situations. This problem suggests that more should be done to train professionals rather than change the criterion for triage.

But suppose it were the case that healthcare professionals lacked this training. In that case, it is reasonable to suggest that triage decisions require bioethical committees for deliberation and include individuals from various professions. The decisions may have to be carried out quickly and online. Still, given the context of a health emergency, it is reasonable not to follow ethical procedures in the same way as in standard circumstances [12]. As mentioned above, a pandemic can be placed in the realm of emergency ethics, and, thereby, standard procedures for ethical decision-making do not apply [9].

Concerning the argument about social trust, it seems more likely that this would happen if decisions were based on clinical criteria than on publicly available and understood criteria. My intuition is that most people would comprehend that, say, a national chief medical advisor leading the nation during a pandemic receives priority over a standard person because of his/her social role in leading the country during a health emergency. However, the decision to prioritise a person with diabetes over, say, a person with cancer is not clear to the public. That is, clinical criteria are more likely not to be understood by the public and, therefore, potentially perceived as arbitrary [13]; Contrastingly, it is reasonable to expect that nations share some common understanding of who has social value. This is because there are likely to be some more or less universal shared values across cultures co-existing in the same nation [14]. Therefore, a triage decision based on social value is less likely to cause a backlash against health professionals, as the reasons are likely to be understood and shared. Surely, there will always be some disagreement, but it is unrealistic to expect total agreement on these matters.

Finally, the argument whereby the social value criterion can lead to arbitrary decision-making that discriminates some groups neglects to mention that this is also the case for clinical criteria. Note that the likelihood of survival is inextricably linked with ethnicity and social class [15, 16]. Individuals from racialized groups such as Latinos and Black people, as well as the working class, are generally more likely to have less access to healthy food, health services, sports facilities, and so forth [17]. Consequently, these individuals are less likely to survive and need more resources compared to individuals from less discriminated against ethnic groups and higher social classes who, throughout their lives, have had access to health resources and good quality of life. I have recently developed an article with a colleague where we show that life-maximizing approaches to triaging imply a form of racism without racists [3]. Racism without racists is a concept we adopted from Eduardo Bonilla-Silva. He understands racism not as a necessarily intentional form of behaviour but as a reinforcement of power structures that maintain and perpetuate racial inequalities [18–21]. Our point was precise that some forms of triaging criteria maintain and perpetuate racial inequalities in health.

Taking this on board, the mere clinical decision neglects that one’s likelihood to benefit from treatment is already embedded in ethnic and class variables, indirectly making decisions to discriminate against already worse-off collectives. There may be an implicit bias when deciding social value, as those making the decisions may belong to a certain social group and make decisions that favor that group. Nevertheless, suppose bioethics committees that make triage decisions are more diverse in terms of gender, sexual orientation, class, ethnicity, and other important social variables. In that case, it is less likely that such decisions will reflect an implicit bias.

### Why accept social value as a criterion for triage?

Although my main goal is a negative one – to show that the arguments presented by those against the social value criterion are mistaken – I should add some reasons why the social value criterion should be considered. I think that some of the positive reasons for this are related to the fact that the social value criterion works better in all the points raised by critics. Regarding society’s perception, it is easier to agree on a social value than clinical criteria. Every society has what Bhikhu Parekh called ‘operative public values’. That is, all societies have a public political culture that is composed of values shared by most members of different classes, independently of their religion, sexual orientation, and so forth [22]. The minimum values give some common groundwork that society can agree on, which the clinical criteria fail to provide. Clinical criteria fail to provide this not only because they are too technical but because they involve topics that are less likely to meet a consensus. For instance, there is unlikely to be a consensus that people with diabetes should be prioritised over people with hypertension. Contrastingly, very few people would disagree that a pedophile ought not to be given priority over a nurse with no record of being a sex offender and who is actively helping people.

Furthermore, as mentioned, because these decisions are more likely understood, these would less likely undermine trust in the profession. People would know exactly why someone is being chosen, without technicalities confusing the public about the reasons for the decisions being made. It is true that most of the time, people have the same level of moral consideration: most people have the same kind of social value, or at least we do not have sufficient information to distinguish them. But this is a positive aspect of the criterion rather than a negative one: it not only treats most individuals equally but is also helpful for evaluating special cases. That is, this criterion has egalitarian implications which are desirable and, at the same time, have conceptual room for adequate exemptions and special cases. Finally, contrasting with what some scholars contend, the social value criterion does not discriminate against collectives for at least two reasons. One is that the social criterion is grounded on the operative public values, the minimum values everyone tends to share. As such, it does not discriminate against the ideology of individuals across sexual orientations, ethnicities, religions, gender, age, and so forth. It coheres with the values that most people share. On the other, it does not meet the complications that the clinical criteria meet in terms of valuing those who are healthier but also members of the most advantaged groups in society (usually, white, males in the upper classes). In other words, the social value criterion does not imply racism without racists, unlike the clinical triage decisions tend to do [3].

## Conclusion

Some scholars uphold that clinical criteria are the most adequate for making triage decisions and dismiss other kinds of criteria, including the individual's social value. In reply, I have contended that their argument fails in two ways. Firstly, they neglect that many of the objections they raise against the social value criterion are also applicable to clinical criteria. Secondly, they exaggerate the implications of their objections to social value criteria. I have demonstrated that having a higher degree of objectivity with the criteria is possible than they uphold. Further research ought to develop forms of institutionalizing political dialogue to establish criteria for triaging.

## Data Availability

NA.
